# A Specialized Multi-Transmit Head Coil for High Resolution fMRI of the Human Visual Cortex at 7T

**DOI:** 10.1371/journal.pone.0165418

**Published:** 2016-12-02

**Authors:** Shubharthi Sengupta, Alard Roebroeck, Valentin G. Kemper, Benedikt A. Poser, Jan Zimmermann, Rainer Goebel, Gregor Adriany

**Affiliations:** 1 Department of Cognitive Neuroscience, Faculty of Psychology and Neuroscience, Maastricht University, Maastricht, Netherlands; 2 Maastricht Brain Imaging Center, Faculty of Psychology and Neuroscience, Maastricht University, Maastricht, Netherlands; 3 Department of Radiology, Center for Magnetic Resonance Research, University of Minnesota Medical School, Minneapolis, MN, United States of America; 4 Center for Neural Science, New York University, NY, United States of America; University Medical Center Utrecht, NETHERLANDS

## Abstract

**Purpose:**

To design, construct and validate radiofrequency (RF) transmit and receive phased array coils for high-resolution visual cortex imaging at 7 Tesla.

**Methods:**

A 4 channel transmit and 16 channel receive array was constructed on a conformal polycarbonate former. Transmit field efficiency and homogeneity were simulated and validated, along with the Specific Absorption Rate, using $B_{1}^{+}$ mapping techniques and electromagnetic simulations. Receiver signal-to-noise ratio (SNR), temporal SNR (tSNR) across EPI time series, g-factors for accelerated imaging and noise correlations were evaluated and compared with a commercial 32 channel whole head coil. The performance of the coil was further evaluated with human subjects through functional MRI (fMRI) studies at standard and submillimeter resolutions of upto 0.8mm isotropic.

**Results:**

The transmit and receive sections were characterized using bench tests and showed good interelement decoupling, preamplifier decoupling and sample loading. SNR for the 16 channel coil was ∼ 1.5 times that of the commercial coil in the human occipital lobe, and showed better g-factor values for accelerated imaging. fMRI tests conducted showed better response to Blood Oxygen Level Dependent (BOLD) activation, at resolutions of 1.2mm and 0.8mm isotropic.

**Conclusion:**

The 4 channel phased array transmit coil provides homogeneous excitation across the visual cortex, which, in combination with the dual row 16 channel receive array, makes for a valuable research tool for high resolution anatomical and functional imaging of the visual cortex at 7T.

## Introduction

At clinical MR field strengths up to 3T, head optimized Radio Frequency (RF) coils tend to be receive-only designs which utilize larger RF body coils hidden behind the bore liner for homogeneous transmit field generation. This setup typically allows for excellent accessibility and space in the system bore, e.g. for fMRI task presentation capability. At ultra high fields, however, the RF wavelength nears the size of the object and RF penetration and interference patterns become a dominant concern [1]. This leads to non uniform *B*_1_ field patterns and a significant difference between transmit and receive *B*_1_ field profiles [2–4]. In this regime, standard RF body coil technology based on either quadrature birdcage or transverse electromagnetic (TEM) designs fail to achieve the required homogeneity and transmit efficiency, and thus RF transmit body coils are not implemented in commercially available Ultra High Field (UHF) scanners. Consequently at 7T it is required to either build a dedicated larger RF transmit coil and combine this with a receiver only array [5–8], or to utilize transceiver arrays [9–11]. Functional images of the human brain at ultra-high fields of 7T and above are often acquired using a cylindrical quadrature volume transmit (Tx) coil and whole head phased array receive coil. Though such a fixed phase coil allows for a high $B_{1}^{+}$ value at the center of the head, the Tx efficiency is significantly lower along the periphery and inferior extents of the brain, particularly along the occipital and inferotemporal visual cortex. Higher transmit efficiency can be achieved with transmit arrays which allow for greater $B_{1}^{+}$ control through RF shimming methods. For fMRI applications that do not require whole head coverage and allow a more focused approach—such as studies of the visual and auditory cortex—it is possible to achieve very high resolution even with a more limited number of receive coils [12]. Both these issues can be addressed by a) using a phased-array, semi-cylindrical Tx coil layout, where $B_{1}^{+}$ can be homogenized by phase and amplitude shimming in the region of interest [13], and by b) additionally utilizing overlapping arrays of small receive coils with closely attached preamplifiers at 3T [14] and 7T [15] in the same RF coil setup.

For fMRI applications cylindrical transmit coils close to the head significantly limit the ability for task presentation even in a (60cm diameter) body gradient setup. Task presentation is even more difficult if high performance head gradient inserts are used for higher spatial and temporal resolution imaging and the available space for coil housing and task presentation hardware decreases to around 36 cm. For human visual system fMRI studies, both in the body as well head gradient setting, coil housing designs which are open at the anterior patient side have significant advantages in terms of fMRI task presentation ability as they easily allow both goggles or mirrored projection setups with large field of view. This is particularly important for ultra-high resolution studies of the visual cortex where both MRI data quality and stimulus presentation quality are of the utmost importance.

If transmit arrays are used in addition to the multiple receive elements, the phase and magnitude of the individual transmit elements can be adjusted (i.e., B1 shimming) to provide an optimized transmit field. The optimized transmit field might entail uniformity throughout the brain, or it might be adjusted to provide maximum efficiency in a more targeted region of interest. Transmit arrays can mitigate Specific Absorption Rate (SAR) issues at high fields [16], as they can be designed to achieve maximum efficiency for the limited regions of interest and can also capitalize on *B*_1_ shimming techniques.

On the receive side, because coil sensitivity profiles are used for spatial encoding, it is important for the receiver layout to comprehensively cover the volume under investigation. Earlier work in phased-array receiver coil design established the benefits of using close-fitting, small diameter surface coils for image locations close to the coil surface [17]. Wald and others [18–21] showed that by incorporating denser receiver coil matrices over the same total surface area, the sensitivity profile of the coil array could be increased substantially for locations close to the coil array surface, ideally without compromising sensitivity at larger distances into the imaging volume. Their analysis predicts that with the increase in the number of receiver channels, it is possible to increase the sensitivity near the array without compromising or losing the sensitivity at distances further away from the coil plane [14].

Here we evaluate the advantages of building an open, half cylindrical Tx coil layout with a conformal small loop size receive (Rx) coil layout optimized for functional MRI of the occipital and inferior and middle temporal visual cortex at high spatial resolution and compare this with a standard whole head array. For the initial half volume coil work we limited ourselves to a 16 receive channel layout for a fairer comparison to the 32 channel whole head array. The coil geometry is chosen such that the receiver coils maintain the critical overlap between neighbouring coil elements, thus minimizing mutual inductance while covering the entire occipito-temporal visual cortex, with very high SNR in the cortex. The coils are mounted on a close-fitting former [22] to maintain close proximity between subject and the receive array. For maximal image SNR, acceleration, SAR reduction, and subject access (i.e. visual presentation, motion monitoring cameras or motion reducing bite bars) an open coil as well as a combination of transmit and receive arrays is desired.

## Methods

### Coil design

The coil was constructed on a 3D printed former made from polycarbonate (PC) using the Fused Deposition Modelling (FDM) (Virtumed LLC, Minneapolis, MN, USA) method and consists of 2 modular parts: one for dedicated receive and one for dedicated transmit function.

**Fig 1:**
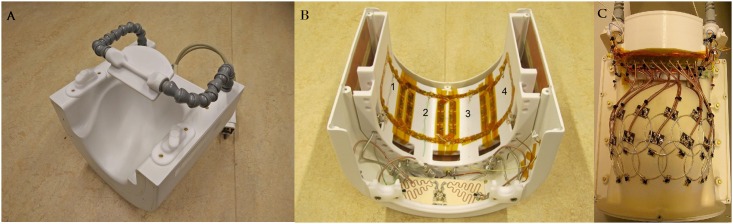
Coil layout. 7 Tesla close fitting dedicated visual cortex coil for high resolution imaging. (A) Shows the assembled coil with provisions for wide field-of-view task presentation mirror (B) The 4 channel phased-array transmit coil with a dedicated power splitter (C) Shows the 2x8 receive coil layout, cable routing and the preamplifier box.

**Fig 2:**
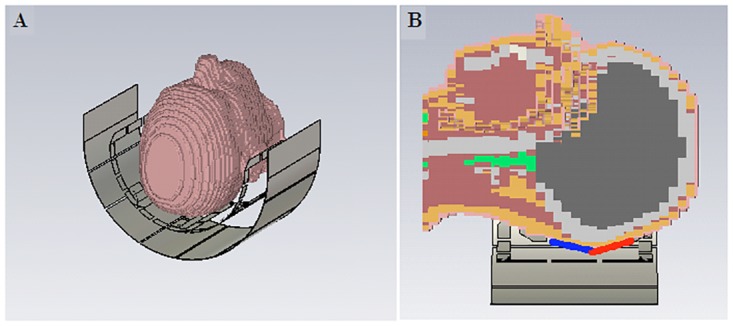
Transmit and receive setup. (A) Design of the 4-channel phased array transmit coil with an RF shield, showing the placement of the subject relative to the coil. (B) The 2 receiver coil rows, indicated in blue and red, encapsulate the human visual cortex.

**Fig 3:**
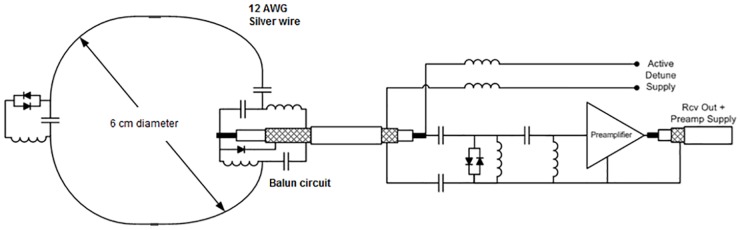
Receive coil schematic. Design of a single receiver coil with the balun circuit, split capacitors, passive and active detuning and preamplifier attached with a *λ*/4 coaxial cable.

16 receive coils were built on a conformal former that was modeled after an average-sized human head (Fig 1A). The inner receive array former is 3 mm thick to ensure minimal distance between the coil and the anatomies under interest, thus maximizing higher receive signal sensitivity. The receive coil layout was designed to ensure maximum SNR in the visual cortex, extending from early visual areas (V1-V4) anterior into occipital, inferior and lateral temporal cortex. Given that the 2 rows of receive coils encapsulate the region of interest in an arc, the coil was named as “Visual Arc”. The receive coils were constructed using 2 mm thick silver-coated copper wires—with each individual coil being 6 cm in diameter—and are laid out in a 8x2 matrix of 16 coils that encapsulate the human occipito-temporal visual cortex (Figs 1C and 2B). A critical overlap (of 0.20 to 0.25 times the loop diameter of each coil; curvature of the receive former dictated the optimal overlap in case of particular coil elements) [17] between all coils was maintained to minimize mutual inductance between coils and to ensure mutual decoupling. Teflon tubing was inserted over each coil such that the overlapping areas of each coil pair would be sufficiently insulated from each other. Two distributed capacitors for each circular loop were divided symmetrically along the coil loop. A detailed schematic of the individual receive coil element is shown in Fig 3.

A 4-channel half-circle phased array transmit coil was constructed concentric to the phased array receive circuit using 1.2cm wide adhesive copper tape (3M, Minnesota, USA) on a half-cylindrical, 3D printed former (Stratasys, Eden Prairie, MN, USA). Fig 2 shows a representation of the placement of the subject, relative to the transmit array and also the placement of the 2 rows of receive coils and their proximity of the sample under investigation.

### Coil circuitry

Each receive coil was connected to a lattice-balun or LC balun circuit [21], [23] and a tuning and matching network comprising of high-voltage ceramic trimmer capacitors *C*_*T*_ and *C*_*M*_ (1-10pf, Johanson Technology, CA, USA) for tuning the coil to resonance at 7 Tesla and matching the coil’s output impedance to a noise match of 50 Ohms. Using small FR4 boards, passive detuning circuitry consisting of an LC circuit in parallel with a high voltage diode was placed on the loop, opposite to the balun circuit. Each coil was tuned and matched to 297.2 MHz and connected to a low input-impedance preamplifier (WMA7TRA, WanTcom Inc., MN, USA) using ∼λ/4 (170mm) length coaxial cable (Huber-Suhner, K02252D), to achieve preamplifer decoupling between individual coil elements by transforming the high impedance at the coil output to the corresponding low impedance at the preamplifier input [17], [23]. The balun circuit incorporates an active detuning circuit across the matching capacitor, using the balun inductor and a PIN diode. When forward biased, the parallel resonant LC circuit adds a high impedance in series with the coil loop, effectively blocking current flow in the receive loop during transmit. The cable length was adjusted carefully for each coil to achieve preamplifier decoupling without requirement of further lumped element phase shifting circuitry. The cables of the lower row were then routed along the center of the upper row coil loops (virtual ground plane) such that there was minimal influence on the reflection and transmission parameters of two adjacent elements in each row.

The preamplifiers themselves were arranged in a 8x2 matrix mirroring the receive coil layout and are placed on a “preamplifier motherboard” which contained the required circuitry for providing the PIN diode voltages for actively detuning each receive coil element during transmit. The preamplifiers were also arranged along the *B*_0_ field or the z-direction of the magnet to minimize any Hall effect which might affect the field effect transistors (FETs) used in these preamplifiers [24]. Cable traps on the output of each preamplifier were required and implemented—by fashioning a solenoid (2 turns, 4mm diameter) from the coaxial cable connecting the preamplifier output to the plug that connects to the magnet bed and bridging a variable capacitor across its ends and tuning it to the resonant frequency of 297.2MHz. Neighboring cable traps were oriented perpendicular to avoid interactions. The traps were all positioned outside the immediate RF transmit loop locations. The traps significantly reduced common-mode currents and were found to be essential to suppress interactions with the transmit coil.

**Fig 4:**
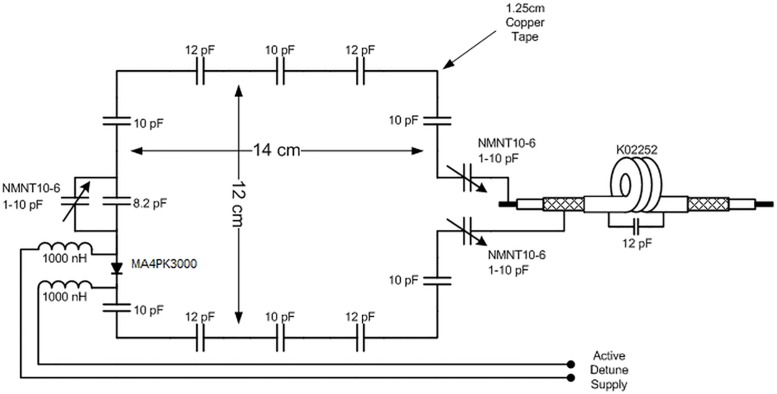
Transmit coil schematic. Design of a single transmit coil with distributed capacitors, tuning and matching capacitors and active detuning.

Of the 4 coil elements of the transmit array, the two peripheral coils are 12x12cm^2^ and contain ten 12pf capacitors distributed along the coil length, while the two central coils are 12x14cm^2^ with four 12pf and six 10pf distributed along the coil length, thus ensuring an extended coverage of the occipital pole and cerebellum (Figs 1B and 2A). A Wilkinson power splitter was implemented at the transmit input to split the transmit signal into 4 equal amplitude excitations for each transmit coil. Each loop was initially phase-shifted by 45° relative to the previous loop by using cables of differing phase lengths, thus ensuring a 135° phase shift between the farthest transmit elements. An RF shield was placed concentric to the transmit array, 3cm away from it. The shield was designed from double sided 50*μ*m thin copper sheet with 8 etched overlaps to minimize eddy current losses. Each transmit loop was tuned and matched to 297.2 MHz and 50 *Ω* respectively using high-voltage variable capacitors (NMNT10-6E, Voltronics Corp, MD, USA), with high-power PIN diodes (MA4PK3000, Macom, MA, USA) connected to each coil in order to detune the transmit array during receive. (Fig 4)

### Bench measurements

Bench tests were performed using an Agilent HP E5071C ENA Series network analyzer, a customized coil-plug bed and a human torso-shaped phantom (49.8% demineralized water, 48.8% sucrose, 1.3% KCl, 0.10% Dowicil; Max Planck Institute for Biological Cybernetics, Tübingen, Germany) as load. Measurements were performed to check for loaded (*Q*_*L*_) and unloaded (*Q*_*U*_) Q ratios, receive coil decoupling and preamplifier decoupling for each coil element. The measurements on the bench validated coil tuning and matching, preamplifier decoupling for each coil element and active coil detuning.

The coil quality factor ratio (*Q*_*U*_/*Q*_*L*_) was measured for a single receive coil element using a dual-loop decoupled (~50dB) inductive probe lightly coupled to the coil loop under test: once as a single element outside the receiver array and once within the populated array while keeping all other receive elements in a detuned state.

Each loop on the receive array was tuned to the corresponding Larmor frequency of 297.2 MHz and matched to 50 Ω. Coupling between neighbouring elements was additionally measured through S21 measurements by directly connecting the coil outputs to the network analyser, while keeping all other elements detuned. Using this setup, the overlap between neighbouring elements could be further optimized to ensure minimal mutual inductance.

Preamplifier decoupling of a single loop was measured as the change in the S21 measurement using a pair of deoupled pickup probes, when the coil element was first power matched to 50 Ω under load but without the preamplifier present and when the coil was terminated using the powered-on low input impedance preamplifier, while all other coils in the receive array were detuned. The active detuning for each receive element was measured as the difference in an S21 measurement, using a pair of decoupled pickup probes, between when the coil is matched to 50 Ω and when detuned.

The assessment of the characteristics of the 4-channel transmit loop was undertaken in the same manner. Each loop of the transmit channel was tuned and matched to 297.2 MHz and 50 Ω respectively using an S11 measurement, with the coil connected directly to the network analyser while loaded with the human-torso phantom. The geometric coupling between adjacent coil elements was measured as the S21 between the outputs of the loaded coil. Active detuning of each coil was recorded as an S21 measurement using a pair of decoupled pickup probes lightly coupled to the coil under investigation, and forward biasing the diode in order to detune the coil. Each coil was terminated to 50 Ω during this measurement, while keeping the other 3 loops in the detuned state.

### EM Coil simulation

The 4—loop phased array transmit coil was simulated in CST (Computer Simulation Technologies, Darmstadt, Germany) in the time-domain, to determine the excited $B_{1}^{+}$ field strength and homogeneity, and to also validate the coil for Specific Absorption Rate (SAR) limits [25]. The coil elements were defined using the software’s in-built 3D CAD designer and included all 4 transmit elements as perfect electrical conductors, with 2 discrete and 8 lumped element ports on each transmit coil. The coil elements were all precisely modeled to accurately reflect the physical design of the coil, including the dimensions and material properties of the elements. The 2 mm diameter wire used for connecting overlapping segments of each coil was also modeled into the simulation setup. The discrete ports allowed us to place variable tuning / matching capacitors and 50 Ω ports in the simulated coil schematic along with predefined capacitors (RLC, series) placed at the lumped element ports along each coil.

The simulation paradigm was completed by placing a human voxel model extended upto the shoulders (Gustav, HUGO voxel family, CST, 2mm isotropic resolution) as the load for the transmit coil and defining a high-resolution mesh (> 50 million mesh cells) around the coil and load. The receive array, along with cable traps, coaxial cable lines and PIN diode detuning cable lines were excluded from the simulation to avoid complex 3D meshing and to save processing time. SAR maps were generated using the electric and magnetic field monitors available through the software and were averaged to 1g and 10g volumes over the voxel model’s region of interest.

### MRI Data Acquisition and Reconstruction

Data was acquired on a Siemens MAGNETOM 7 Tesla actively-shielded MRI system (SIEMENS Healthcare, Erlangen, Germany) with an SC72 gradient coil capable of a maximum gradient strength of 70mT/m with a maximum slew rate of 200T/m/s. Data pertaining to coil characterization, namely tSNR, g-factor maps and noise correlation matrices were obtained through in-vivo scans across both the Visual Arc and a 32 channel whole head coil (Nova Medical,Inc., MA, USA) for two healthy subjects. Subjects for the study filled out a written consent form prior to imaging, as approved by the Ethical Review Committee Psychology and Neuroscience (ERCPN) of Maastricht University.

Transmit field or $B_{1}^{+}$ field quantification was achieved by using the Actual Flip Angle method (AFI) [26]. In-vivo tSNR (Temporal Signal-to-Noise Ratio) maps were obtained at two different resolutions using the University of Minnesota’s multi-band EPI package for Gradient Echo BOLD EPI [27] with the following parameters: Echo Time (TE) = 17 ms; Repetition Time (TR) = 2000 ms; Partial Fourier (PF) = 6/8; GRAPPA 3; Bandwidth (BW) = 1488 Hz/Px; Phase-encode BW = 27.5 Hz/Px; field of view (FOV) = 192 x 192 mm^2^ for a 1.2 mm isotropic acquisition and Echo Time (TE) = 23 ms; Repetition Time (TR) = 2000 ms; Partial Fourier (PF) = 6/8; GRAPPA 3; Bandwidth (BW) = 1102 Hz/Px; Phase-encode BW = 18 Hz/Px; FOV = 130 x 130 mm^2^ for a 0.8mm isotropic acquisition. Noise covariance data was acquired using the same pulse sequence with the transmit voltage set to 0 volts, or without an RF excitation.

For phantom datasets the following parameters were used for EPI acquisitions: Echo Time (TE) = 18 ms; Repetition Time (TR) = 3000 ms; Partial Fourier (PF) = 6/8; GRAPPA 3; Bandwidth (BW) = 1394 Hz/Px; Phase-encode BW = 26.5 Hz/Px; FOV = 166 x 166 mm^2^ for a 1.2 mm isotropic acquisition and Echo Time (TE) = 27.6 ms; Repetition Time (TR) = 3000 ms; Partial Fourier (PF) = 6/8; GRAPPA 3; Bandwidth (BW) = 1002 Hz/Px; Phase-encode BW = 13.1 Hz/Px; FOV = 166 x 166 mm^2^ for a 0.8mm isotropic acquisition.

Image SNR for both coils was computed using a pseudo-replica approach [28, 29] from the short-TE GRE image and noise data acquired using the same subject, which is also the data used for g-factor analysis. Noise data was acquired using each coil without RF excitation and the standard deviation and correlation of the noise data were computed. We generated a 100 replicas by adding random samples from the noise scan onto the GRE k-space of the standard image acquisition prior to Fast Fourier Transform (FFT), while maintaining the noise correlation between the receive channels. The final image SNR was then calculated as the ratio of the mean of the image to the standard deviation of the noise over these 100 replicas. A mask covering the brain volume containing the occipital lobe was created to determine SNR values.

tSNR maps were computed as the mean signal intensity over time divided by the standard deviation of the time series, averaged over a 100 volumes after detrending for linear and 1-2 cycle oscillations and up to second order harmonic oscillations. Data analysis was performed using BrainVoyager QX 2.8 (Brain Innovation, Maastricht, The Netherlands) and custom-written routines in MATLAB (The MATHWORKS Inc., Natick, MA, USA). In addition to the MPRAGE and and fMRI scans, we acquired noise-only scans and fully sampled 3D GRE for evaluation of the noise correlation and the parallel imaging performance of the two coils. Both scans (in-vivo and phantom) were performed using a 3D GRE sequence with the following matched parameters: Echo Time (TE) = 2.64 ms; Repetition Time (TR) = 30 ms; FOV = 256 x 256 mm^2^; 2 mm isotropic; the noise scan was acquired with fewer *K*_*Y*_ and *K*_*Z*_ encode and by turning the transmitter voltage to zero. The GRE scan was Fourier-interpolated to a matrix size of [120 x 120 x 120] in order to allow emulation of different SENSE undersampling factors on the bases of data set (R = 2, 3, 4, 5, 6, 8). For a quantitative comparison of the parallel imaging performance of the two coils, noise correlation matrices, coil sensitivity maps and g-maps were calculated in MATLAB.

Sensitivity maps were computed as described by Walsh [30]. For the g-factor maps we used the formulation given in the original SENSE paper [31].

$$g_{\rho }=\sqrt[{}]{{\left[{\left(S^{H}\Psi^{-1}S\right)}^{-1}\right]}_{\rho ,\rho }{\left(S^{H}\Psi^{-1}S\right)}_{\rho ,\rho }}$$ (1)

where Ψ is the covariance matrix. Both g-factors and corresponding SENSE reconstructions were performed at undersampling factors between 2 and 6, by sub-sampling the full k-space data set that had been re-sampled to [120 x 120 x 120]. The reconstruction itself was a SENSE reconstruction as described in [31]. We have chosen SENSE g-maps because we feel that this is more general, but alternatively GRAPPA or pseudo-replica based g-maps could have been shown.

The visual paradigm used for fMRI data acquisition consisted of high contrast flickering checkerboards (flicker rate of 7.5 Hz) as a passive viewing task with central fixation dot on the screen. A block design was chosen with 18 blocks of checkerboards with length 4 TRs (8s) interspersed with rest periods of 5,6, or 7 TRs (jittered). The frontal-open design of the 16 channel coil allowed for a visual angle was 29°—extending into the subjects’ near-peripheral vision; for the 32 channel whole head coil the visual angle was 10°—extending into the subjects’ central and paracentral vision [32]—as its viewing angle is limited due to the coil’s design.

## Results

**Fig 5:**
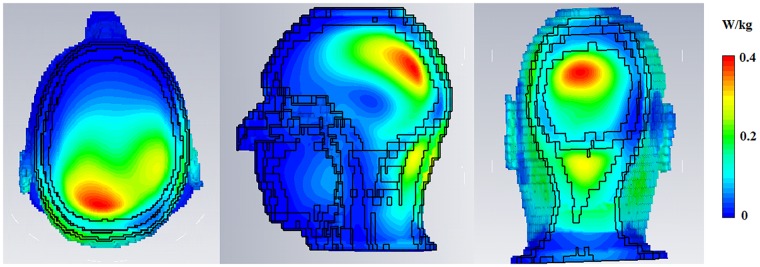
SAR results. Specific Absoprtion Rate or SAR_10*g*_ for the HUGO voxel model as simulated in CST, shown along all 3 axes. Peak SAR_10*g*_ was computed as 0.385 W/kg.

Inter-element decoupling between pairs of transmit elements was -13dB or lower. Active pin-diode detuning of each transmit loop, recorded as an *S*_21_ measurement using a double-loop probe, was about -20dB. Table 1 shows the S-parameter matrix for the 4-channel phased array transmit coil when loaded with the human-torso phantom. Electromagnetic simulations of transmit array for SAR validation yielded a peak SAR value of 0.385 W/kg in the human voxel model for a fixed-phase increment across coils, for a 1 Watt sinusoidal RF excitation split across 4 channels equally through the Wilkinson power splitter, as illustrated in Fig 5. To ensure patient safety, a conservative factor of 1W/kg was chosen as the SAR limit.

**Table 1:** S-parameters in dB showing the transmission and reflection coefficients for the 4-channel transmit coil when loaded with the human torso phantom.

Channel	1	2	3	4
1	-29.8	-13.5	-15.2	-21.4
2		-30.3	-14.2	-16.6
3			-28.2	-14.8
4				-31

**Fig 6:**
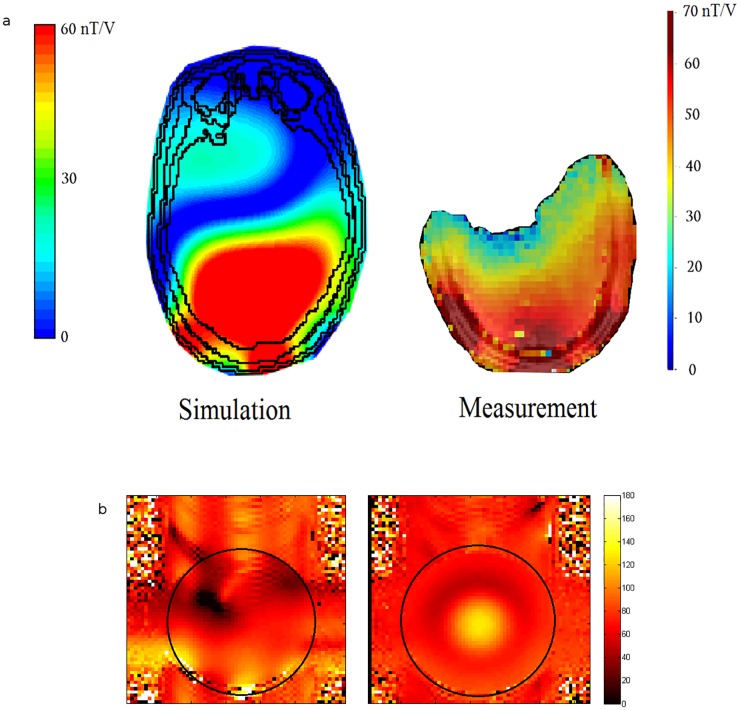
Transmit field maps. (a) Axial slices showing simulated (L) and measured (R) $B_{1}^{+}$ field maps in nT/V for the Visual Arc. (b) Flip angle maps (in degrees) of the Visual Arc (L) and Nova coils (R) using a spherical phantom (represented by the black circle).

Fig 6 shows the simulated and measured $B_{1}^{+}$ maps along a transverse slice for the voxel model and the human subject respectively. The measured $B_{1}^{+}$ data was acquired using a quantitative *B*_1_ mapping method, co-registered to an anatomical image of the same slice in the axial plane. Both simulation and measured data show good correlation between each other, with a substantially homogeneous $B_{1}^{+}$ excitation profile achieved across the visual cortex.

**Fig 7:**
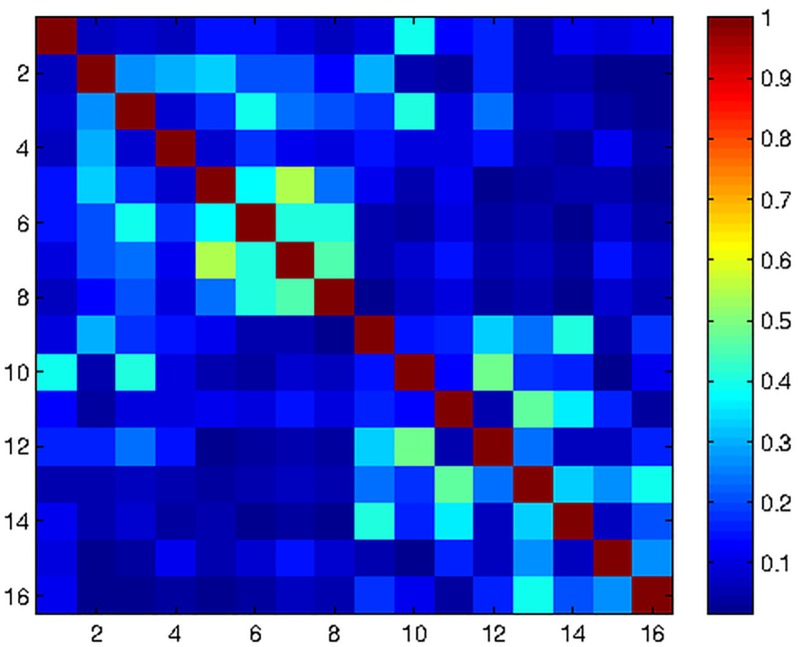
Noise correlation. Noise correlation matrix for the constructed 16 channel Visual Arc coil, with a peak of 54% and a mean of 19%.

The unloaded Q factor *Q*_*U*_ (indicative of coil losses) for an isolated receive element was about 240 and the loaded Q factor *Q*_*L*_, indicative of coil and tissue losses, was 25, giving us a *Q*_*U*_/*Q*_*L*_ of about 9.6, indicating sample noise dominance. For a single receive loop surrounded by 5 detuned loops in the array, the same Q factor measurement yielded a *Q*_*U*_/*Q*_*L*_ of 8.6 (232/27). All receive coils were matched between -19dB and -23dB for the human torso phantom and for upto 3 different subject-head sizes. The active PIN diode detuning provided an isolation better than 25dB between tuned and detuned states. The decoupling between neighbouring, overlapping receive elements ranged between -12dB to -18dB. Decoupling between next-nearest neighbouring elements (or non-overlapping elements) of the array ranged between -15dB to -24dB. Preamplifier decoupling accounted for an additional 20dB of isolation. The noise correlation matrix, as shown in Fig 7, was obtained with in-vivo scanning, with the correlation ranging between 0.2% to 54% with an average of 19%.

**Fig 8:**
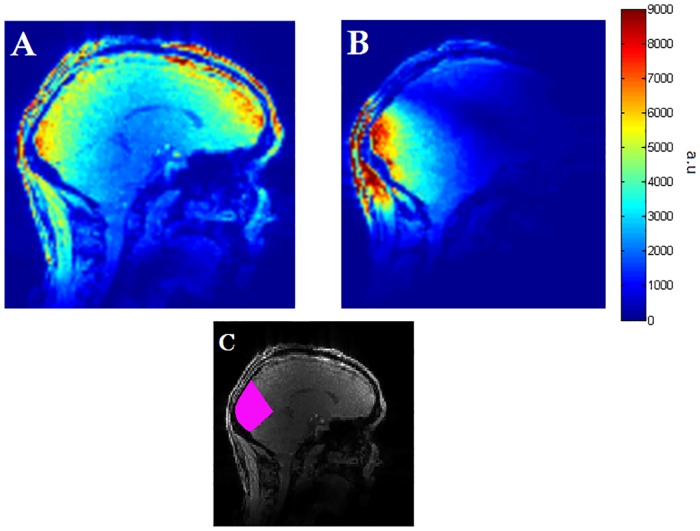
Signal-to-Noise comparison. (A) SNR map of the 32 channel head coil with a human subject (B) SNR map of the 16 channel Visual Arc coil, with the same colour scale used for both maps. (C) Mask (in pink) showing brain volume used for SNR calculation.

**Fig 9:**
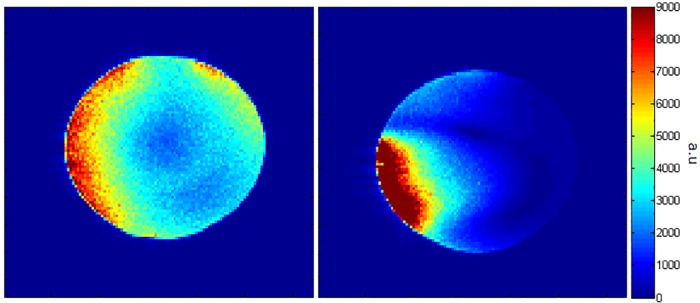
Signal-to-Noise comparison. (L) SNR map of the 32 channel head coil with a spherical phantom and (R) SNR map of the 16 channel Visual Arc coil, with the same colour scale used for both maps.

Image SNR for both coils is shown in Fig 8 for in-vivo scans and Fig 9 for a spherical phantom. The mean SNR in the masked volume was 5318 for the 32 channel whole brain coil and 7139 for the Visual Arc coil, while the maximum SNR values in the same volume were 6988 and 10014 for the whole head coil and the Visual Arc coil respectively.

**Fig 10:**
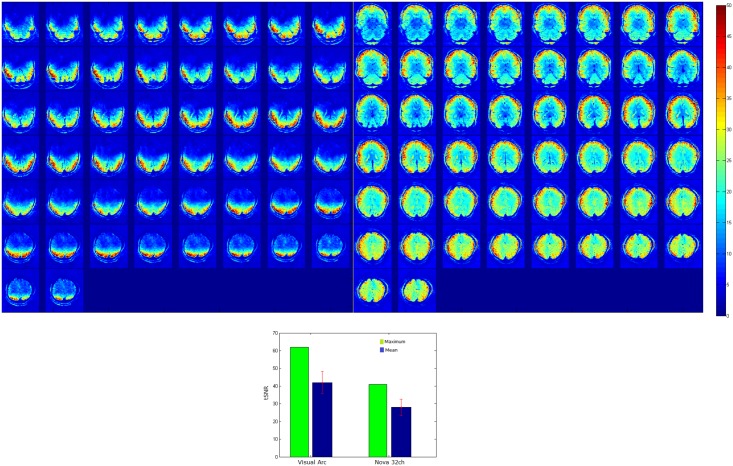
tSNR maps at 1.2mm isotropic, for an transverse oblique slice stack along the visual cortex for the (left) Visual Arc coil and (right) the 32 channel whole head coil. (bottom): bargraph showing tSNR comparison at 1.2mm isotropic between the 16 channel visual coil and the commercial 32 channel Nova coil.

**Fig 11:**
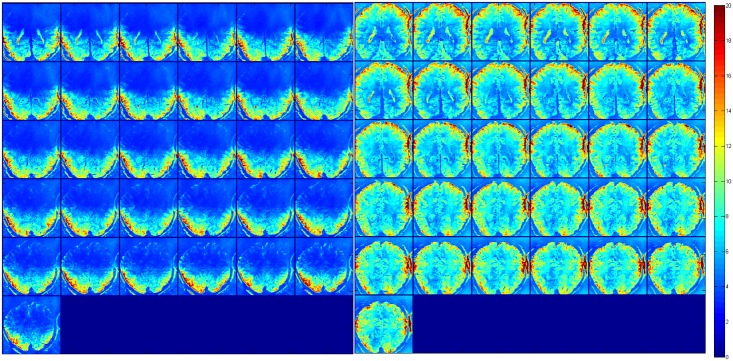
tSNR maps at 0.8mm isotropic, for an transverse oblique slice stack along the visual cortex for the (left) Visual Arc coil and (right) the 32 channel whole head coil.

**Fig 12:**
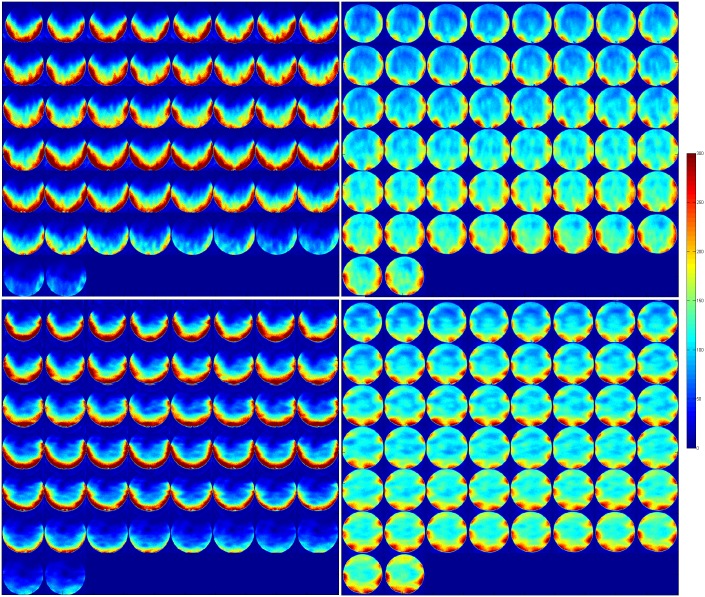
tSNR maps at 1.2mm isotropic, for an transverse slice stack using a spherical phantom for the (left) Visual Arc coil and (right) the 32 channel whole head coil using L-R phase encoding (top) and A-P phase encoding (bottom).

**Fig 13:**
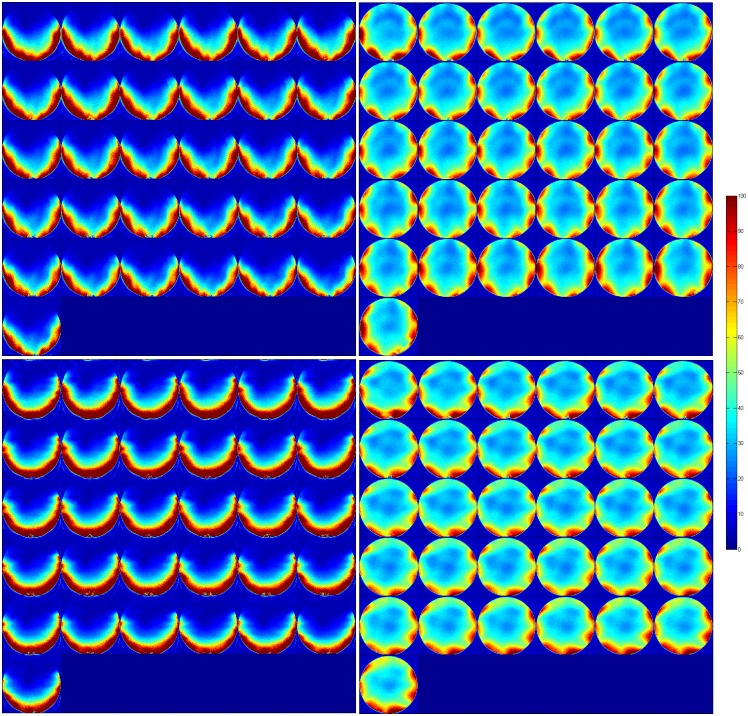
tSNR maps at 0.8mm isotropic, for an transverse slice stack using a spherical phantom for the (left) Visual Arc coil and (right) the 32 channel whole head coil using L-R phase encoding (top) and A-P phase encoding (bottom).

**Fig 14:**
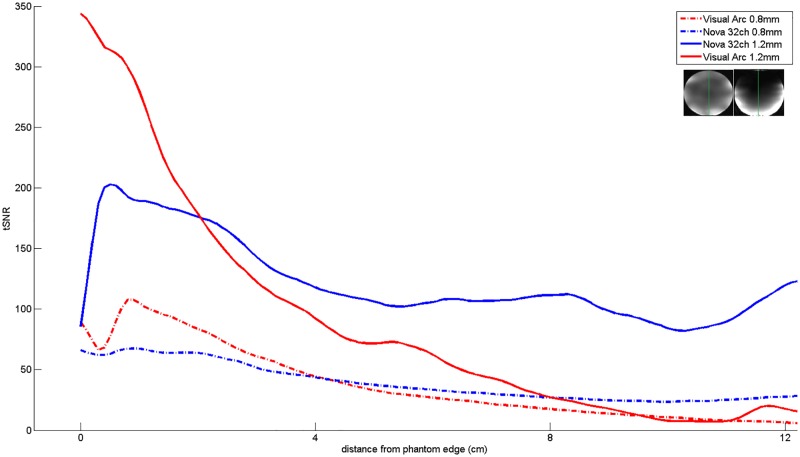
tSNR profiles with respect to imaging depth. The green line (inset) represents the slice along which the tSNR profiles were plotted.

Temporal SNR maps were generated for both the 16 channel visual coil and the standard 32 channel whole head coil at 1.2 mm isotropic resolution using a gradient echo—EPI sequence and the same measurements were repeated with a spherical phantom. A small region of interest covering the primary and secondary visual areas (V1 & V2) was defined and maximum and mean tSNR values were computed in the same ROI. Fig 10 shows the maximum and mean tSNR value estimates generated by both coils in the primary and secondary visual areas at 1.2mm isotropic. tSNR was about 1.5 times higher with the 16 channel Visual Arc coil (than with the 32 channel whole head coil), with the high tSNR receive profile extending into the inferotemporal cortex. The receive array provides high SNR and sufficient penetration depth to image the visual cortex into sulcal depths. Fig 11 shows the tSNR estimates for the same subject at 0.8mm isotropic resolution. Figs 12 and 13 shows the entire slice stack of the phantom EPI measurement, acquired using both left-right and anterior-posterior phase encoding. tSNR profiles along a central slice were plotted for both coils at 1.2 and 0.8mm isotropic using a spherical phantom. Fig 14 shows the tSNR profiles as a function of distance from the edge of the phantom.

**Fig 15:**
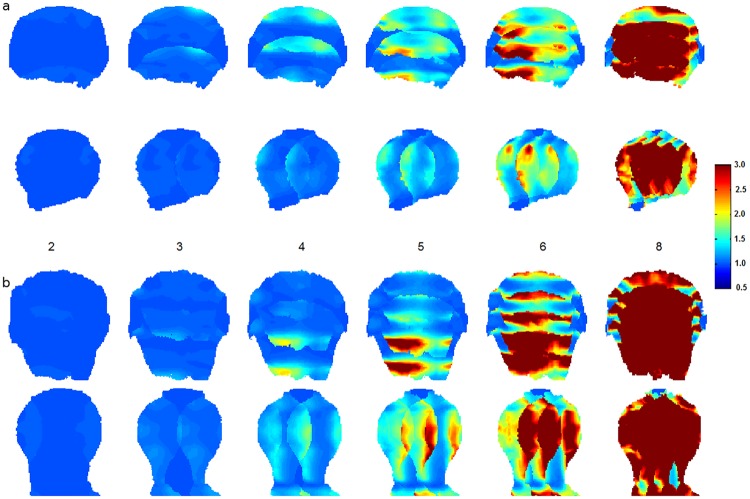
g-factor maps. Coronal g-factor maps for the (a) 16 channel Visual Arc coil for S-I and L-R accelerations respectively and (b) the standard 32 channel whole brain coil for acceleration factors R = 2,3,4,5,6 and 8.

**Fig 16:**
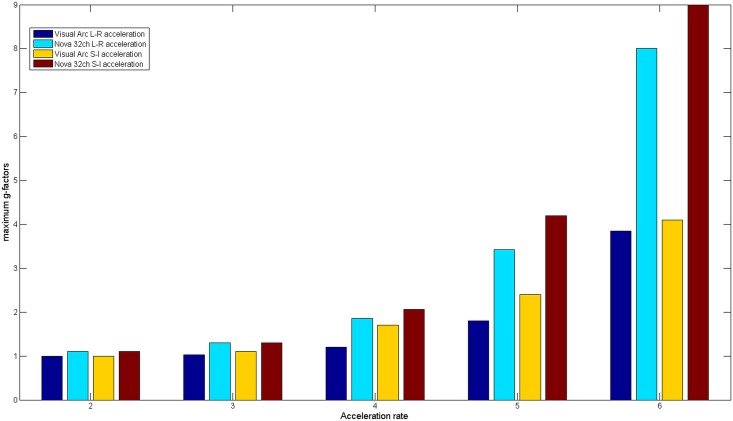
Maximum g-factors. Maximum g-factors as a function of the acceleration rate, plotted for the 16 channel Visual Arc coil and the standard 32 channel whole brain coil.

**Fig 17:**
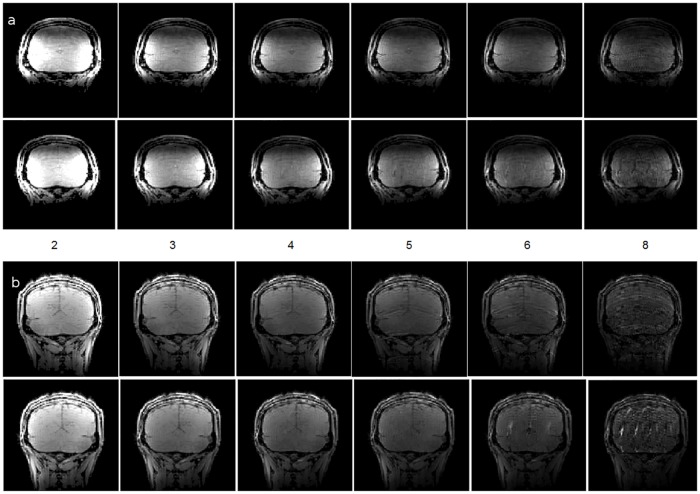
SENSE Reconstruction. SENSE reconstructed images from the (a) Visual Arc coil for A-P and L-R accelerations respectively and (b) the 32 channel whole head coil for acceleration factors R = 2,3,4,5,6 and 8 for the same coronal slice across both coils.

Local g-factor maps in a coronal plane were generated for both the 16 channel visual coil and the 32 channel whole head coil for one dimensional accelerations, both left-right and superior-inferior, using data from coil sensitivity profiles and noise correlations as acquired from subject measurements. g-factors for the Visual Arc coil were lower than those for the 32 channel whole brain coil for all acceleration factors, as seen in Fig 15. At R = 4, the maximum g-factor for the for the 32 channel coil was 1.5 times that of the Visual Arc coil and almost 3 times as much at R = 6, as plotted in Fig 16. Fig 17 shows the SENSE reconstructed images for both coils.

**Fig 18:**
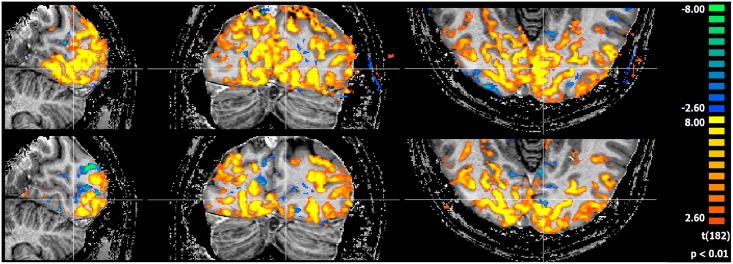
fMRI at 1.2mm isotropic. fMRI activation maps along 3 planes for the 16 channel visual coil (top) and the 32 channel head coil (bottom) at 1.2mm isotropic.

**Fig 19:**
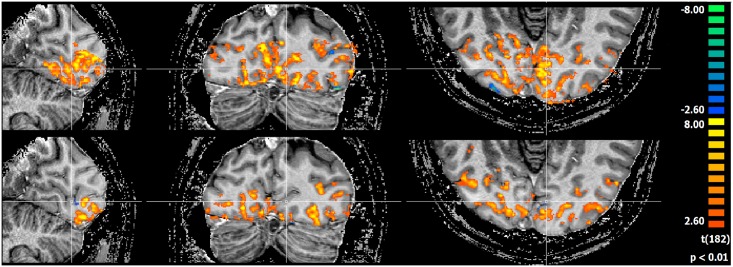
fMRI at 0.8mm isotropic. fMRI activation maps along 3 planes for the 16 channel visual coil (top) and the 32 channel head coil (bottom) at 0.8mm isotropic.

Functional MRI data was acquired at 1.2mm (Fig 18) and 0.8mm (Fig 19) isotropic resolution using both coils, as shown in the activity maps. The maps are thresholded to the same t-value of 3.5 (p<0.01) across both coils and both resolutions. Through a combination of a wide visual angle and densely packed receiver coils in the region of interest, the Visual Arc coil shows activation deeper into the occipital lobe, both in the superior-inferior and anterior-posterior directions at 1.2mm resolution. The resulting activation signals at 0.8mm isotropic resolution conform to a similar pattern, indicating that notwithstanding the small voxel size, fMRI responses can be measured adequately if appropriate imaging hardware are used.

## Discussion

In this study, we constructed and characterized a semi-cylindrical phased-array transmit coil and a shape-optimized, 16 channel receive coil targeted at acquiring high-resolution anatomical and functional MRI data at 7T. The coil’s performance was evaluated through bench tests, phantom and in-vivo imaging. The close fitting transmit coil does not require otherwise time-consuming tune and match adjustments for different subjects, lowering experimental setup time. The placement of the 2 rows of receive arrays not only covers the entire visual cortex, but also lies conformal to it, making sure that the coils are as close as possible to the region of interest and enhancing their receive sensitivity when compared to that of the 32 channel whole head coil. We did observe a slight frequency shift between loaded and unloaded cases, which was compensated for by using the human-torso shaped phantom during bench tests. Differences were also observed between the simulated and measured $B_{1}^{+}$ transmit maps, which can be attributed to the shape and positional placement differences between the in-vivo subject and the voxel model used for simulation.

It is also noted that the *B*_1_ transmit and receive profiles show the expected asymmetry at high fields while maintaining a homogeneous excitation in our targeted ROI (namely the primary and secondary visual areas), as is the case here. One way of countering this issue is by shifting the transmit and receive coils or arrays in opposing directions, by the same angle, along a circular or curved former [33], [8]. Although this would have been possible to achieve with the transmit coils on the Visual Arc, the corresponding shift of the receive array would only be possible with an unsymmetrical holder layout [Fig 1C], leading to more restricted visual openness. We did not pursue this option since we aimed to maintain full presentation capabilities but would consider it for future work.

The mean noise correlation between receive coil elements was a respectable 19%; however, the worst-case correlations of 54% were found in adjacent coil elements in the same column. This high correlation is due to (1) insufficient overlap and thus decoupling between loops that traverse the curved surface of the former and (2) due to shared resistance acquired through the sample.

The image SNR measured shows a 1.35 fold increase in mean SNR and a 1.44 fold increase in maximum SNR for the Visual Arc coil over the standard head coil (in the occipital lobe), and this can be attributed to a combination of a tight-fitting former and smaller coil loops. This is further evident in the tSNR maps, whereby we are able to achieve sub-millimeter resolutions, allowing us to image the visual cortex into sulcal depths. The g-factors for the Visual Arc were substantially lower than those for the 32 channel whole head coil, especially at acceleration factors intrinsically associated with fMRI data acquisition (R = 3, 4).

Previous studies involving smaller FOV’s covered by extremely high density 16 channel phased-array layouts of 1x2cm^2^ small coils at 7T have been reported by Petridou et al [15]. While we did not include such a small FOV array in our comparison, such arrays are expected to and do have very high SNR for cortical structures in the immediate vicinity of the coils. We however pursued a path of using larger coils capable of a more extensive FOV because of the limited coverage and the inherent rapid fall-off of extremely small loops. Nevertheless, we expect that in the future high density arrays covering perhaps even larger cortical regions with channel counts beyond 32 will play a significant role in high resolution fMRI.

For fMRI tests, the combination of a wide-angle subject field-of view (due to the frontal-open design of the Visual Arc) and conformal, small receive coils allow for efficient visual task presentation and increased sensitivity to BOLD response, allowing for sub-millimeter fMRI acquisition. The added activation on the anterior side is due to the open design of the coil, as this cortical region represents more eccentric retinotopic positions. We even notice negative activation with the 32 channel whole head coil in the same regions. They reflect a well known phenomenon of lateral inhibition which causes negative BOLD adjacent to stimulated areas [34].

The benefits of the 4 channel transmit array could be further enhanced by incorporating parallel transmit hardware that would allow for better control over $B_{1}^{+}$ fields and possibly help reduce SAR effects even further. At the receive end, it is abundantly clear that denser coil arrays would be beneficial for human brain imaging, ideally using coils of an optimised diameter (likely around 4 cm) at 7T. Future work would involve increasing the number of receive channels beyond 32 and towards high channel counts (96 and even 128, as already presented by Wiggins et al [14] at 3T) while increasing the overall volumetric coverage to be able to image the entire human brain.
